# Anxiety and Depression Levels in Parents after Counselling for Fetal Heart Disease

**DOI:** 10.3390/jcm12010394

**Published:** 2023-01-03

**Authors:** Gizem Seyda Erbas, Christoph Herrmann-Lingen, Eva Ostermayer, Alexander Kovacevic, Renate Oberhoffer-Fritz, Peter Ewert, Annette Wacker-Gussmann

**Affiliations:** 1 German Heart Center, Department of Pediatric Cardiology and Adult Congenital Heart Disease, 80636 Munich, Germany; 2 Department of Psychosomatic Medicine and Psychotherapy, University of Göttingen, Medical Center and German Center for Cardiovascular Research (DZHK), Partner Site Göttingen, 37075 Göttingen, Germany; 3 Department of Obstetrics, Klinikum Rechts der Isar, 81675 Munich, Germany; 4 Department of Pediatric and Congenital Cardiology, Heidelberg University Hospital, 69120 Heidelberg, Germany

**Keywords:** depression, anxiety, fetal cardiology, counselling, fetal heart disease, antenatal diagnosis

## Abstract

The progress in fetal cardiology allows for the early diagnosis of congenital heart defects, but there is still a lack of data on the psychological situation of parents expecting a child with a congenital heart defect. In this cross-sectional study, 77 parents (45 women and 32 men) expecting a child with a heart defect were interviewed with different questionnaires. The standardized Hospital Anxiety and Depression Scale (HADS) questionnaire was used to assess the psychological state of the parents. Various statistical procedures were performed to determine the prevalence, risk factors, and predictors of anxiety and depression. The prevalence for prenatal anxiety was 11.8% and for depressed mood 6.6%, whereas the postnatal prevalence was 25% for anxiety and 16.7% for depressed mood. The mother is influential in protecting against depression as a contact person (*p* = 0.035). Women were more affected by anxiety and depression than men (*p* = 0.036). A significant and positive correlation was observed between anxiety and depression before birth (ρ = 0.649, *p* < 0.001) and after birth (ρ = 0.808, *p* < 0.001). The level of education correlated negatively with depression (*p* = 0.016) and anxiety (*p* = 0.017) before birth. Significantly higher anxiety and depression scores were not observed among health and social workers (*p* = 0.084), first-time mothers (*p* = 0.190), and parents whose pregnancies were due to medical assistance (*p* = 0.051). Close collaboration between maternal-fetal care units, pediatric cardiologists and psychiatric/psychosomatic disciplines is a possible strategy to reduce stress in parents. Therefore, an expert team of professionals, educating with understandable terms and sufficient knowledge about fetal heart disease in parenting counseling, is required. The support of affected parents can positively impact the treatment of the child and should be integrated into the daily routine of the clinic.

## 1. Introduction

Heart defects are the most common congenital malformations in humans with an incidence of 75 per 1000 live births including all forms of heart diseases [1]. Studies showed that both the early detection of a congenital heart defect in pregnancy and the accuracy of the diagnosis improved significantly from 1996 to 2013 [2]. This is a result of medical and technical advances in obstetrical care over the past 20 years and interdisciplinary collaborations [3]. Pediatric cardiologists, pediatric heart surgeons, anesthesiologists, obstetricians neonatologists, geneticists, and psychologists are predominantly involved in fetal diagnostics, parental counselling, and postnatal treatment of the child [4].

Currently, screening examinations are possible from the first trimester onwards; however, the majority of pregnant women receive second trimester screening.

This raises the question of whether prenatal scans and consecutive counselling can lead to anxiety and depression symptoms in the expectant parents.

A systematic review that excluded high-risk pregnancies to provide an overview of general pregnancies showed that the prevalence of any antenatal depression was 20.7% [5], and that anxiety was present in 15.2% [6]. According to Dagklis et al., the prevalence of perinatal depression in high-risk pregnancies was even higher with 28% [7]. Woolf-King et al. showed that parents of children with severe congenital heart defects suffer from mental distress such as post-traumatic stress disorder, anxiety, depression, stress, or other mental illnesses. In particular, the periods before and after the operations of the children are very stressful [8]. In their “research agenda and recommendations”, Sood et al. confirmed that further research is required to optimize screening methods and intervention options for parental psychological distress and its impact on child outcomes [9].

Anxiety and depression during pregnancy are associated with adverse consequences for both parents and the child. Mothers affected by prenatal anxiety and depression showed increased parenting stress postnatally [10]. In addition, the child can be negatively affected by the mother’s emotional state [11], resulting in a shorter gestation period and altered development both in general and at the neuronal level. This manifests itself in a lower birth weight of the child, motor and cognitive disorders, problems at the social and emotional level, and psychological problems in the children [12].

Therefore, counselling sessions before birth by maternal–fetal specialists and cardiologists that follow the diagnosis of a fetal heart defect are essential for parental understanding and further decision making. The physician’s task is to explain the fetal heart defect in a comprehensible way, to outline the necessary measures after birth, and to provide information about the long-term prognosis (including aspects of quality of life (QoL)). This should provide a valuable basis for the parents to deal with this stressful situation [13,14].

The aim of our study was to assess the psychological state of parents expecting a child with a congenital heart defect in order to subsequently improve support services and minimize negative consequences for the mother and the child.

## 2. Materials and Methods

### 2.1. Participants and Study Setting

A longitudinal questionnaire-based study was conducted from October 2019 to April 2021 in the Department of Congenital Heart Defects (German Heart Center, Munich).

The study participants were parents expecting children with congenital heart defects who were counselled for fetal heart disease at our department. The team consisted of fetal medicine specialists, pediatric cardiologists, cardiac surgeons, nurses, and psychologists. If necessary, a genetician could be involved. The individual heart defect of the child was explained in a generally understandable way, and the probable course of the disease was discussed. Pictures were used for illustration, and recommended websites were communicated to the parents. Furthermore, if desired, contact was made with other affected parents. Sufficient knowledge was provided to the parents.

The questionnaire was given to the expecting parents following the consultation or was posted after a telephone consultation due to the pandemic. Written informed consent was obtained from all participants. Parents were eligible for inclusion if they had a minimum age of 18 years and if the child had a prenatally detected heart defect. After contacting 81 families, the questionnaire was distributed to 73 families. Five families declined to participate, three did not wish to participate because they were abroad, and forty-five families completed the questionnaire, with a lower participation rate among men. A total of 77 subjects participated in the study, including 45 mothers and 32 fathers. For a follow-up, 24 participants completed the questionnaire again 5–13 months after the birth of the child. T1 was before birth and t2 after birth.

The study was approved by the ethics committee of the Technical University of Munich No. 387/19S.

### 2.2. Variables and Assessment Measures 

A questionnaire was designed to assess parental symptoms, including personal and sociodemographic data. A validated screening questionnaire for self-assessment of depressive and anxiety symptoms was also used.

The questionnaire consisted of closed questions, including dichotomous and multiple-choice questions. It was created to provide information regarding personal data (sex, age, native language, country of birth), education and profession (current employment relationship, occupational field), socio-demographic information (marital status, living arrangement, details about parental leave), and pregnancy (planning the pregnancy, support via medical measures and social environments). Regarding the professional field, we distinguished between “health, social services, teaching and education” and “the other sector”. In this context, the “other sector” includes agriculture; forestry; animal husbandry and horticulture; raw material extraction; production and manufacturing; construction; architecture, surveying, and building technology; natural science; geography and information technology; transportation; logistics, protection and security, commercial services, commodity trading, distribution, hotels and tourism, business organization, and accounting; law, administration, and language; literature; humanities; social and economic sciences; media; arts; culture and design; and military. In addition, the validated self-completion “Hospital Anxiety and Depression Scale (HADS)” was used to assess anxiety and depression. The questionnaire consisted of 14 items—7 were anxiety-related (HADS-A) and 7 measured depressed mood (HADS-D). The response options consisted of a 4-point rating scale (0–3 points), which resulted in scale values ranging from 0 to 21 for each subscale. Scores ≥11 per subscale were defined as “significant”, indicating the presence of anxiety or depressed mood. Those with scores ≤ 7 were defined as “not significant”, indicating the absence of anxiety or depressed mood. Values of 8–10 were considered to be “borderline” [15,16,17].

In validity studies, the HADS has been shown to be a suitable screening tool for gynecological and pregnant patients [18,19].

### 2.3. Statistical Analysis

To describe the characteristics of participants, all variables were initially evaluated descriptively. The severity of the heart defect was categorized in three subgroups. Two groups were formed depending on the number of children; Group 1 had no children, while the parents in Group 2 had 1 child or more. Spearman correlation analyses, Fisher’s exact tests, and the Kruskal–Wallis tests with post-hoc Dunn–Bonferroni tests were performed to assess the characteristics associated with anxiety and depression.

Changes in the scale scores for depression and anxiety between the two survey time points were examined using the Wilcoxon test for paired samples. Cramér’s V was used as an effect size for tests of association. For Kruskal–Wallis tests, ɛ^2^ was used as effect size and the matched-pairs rank biserial correlation coefficient was used for Wilcoxon tests.

The central component of the statistical investigation was the HADS, in which the questions were summarized to form one score per subscale. 

Another variable was formed to combine anxiety and depression into an anxiety and depression syndrome. To test for differences in depression and anxiety between the two time points, Wilcoxon tests were applied. 

To identify predictors of anxiety and depression symptomatology, multivariate analyses using logistic regressions were performed in a final step. 

SPSS software version 27.0 was used for all analyses.

## 3. Results

### 3.1. Participant Characteristics

In total, data were collected from 77 parents, of whom 45 (58.5%) were female. The mean age of the respondents was 33.70 (SD = 5.262) and the participants ages ranged from 20 to 45 years. The majority of the sample (n = 66, 85.7%) reported “German” as their native language (Table 1).

### 3.2. Education and Profession

In the present data, there was a significant relationship between education levels and anxiety or depression. Individuals with a higher educational level were less affected by depression (Fisher *p* = 0.016, Cramér’s V = 0.276) and anxiety (Fisher *p* = 0.017, Cramér’s V = 0.307) at time t1.

In the group of parents who had no school-leaving qualification or a lower secondary or intermediate secondary school-leaving certificate, the HADS mean score at time t1 was 8.00 ± 3.32 for anxiety and 4.93 ± 3.77 for depression. In contrast, in the group of parents who graduated from university, the mean score was 5.55 ± 2.85 for anxiety and was 2.23 ± 1.92 for depression. In relation to the anxiety and depression syndromes, there was also a significant Fisher test (*p* = 0.005). In the group of depressed parents, 60% had completed an apprenticeship, and 40% had either no school-leaving qualification or a lower secondary or intermediate secondary school-leaving certificate. The group of “not significant” parents was dominated by those with a university degree (44.6%). The situation was similar for parents suffering from anxiety, as 28.6% had no qualifications or a secondary school diploma, 14.3% had a university entrance qualification, 50% had apprenticeship, and 7.1% had completed university. Among the “not significant”, 52.3% had graduated. Individuals with higher degrees were typically less affected.

Furthermore, a marginal association between anxiety/depression at time t1 and the occupational field was also found. The “other sector” was less affected than the “health, social services, teaching and education” sector (Fisher *p* = 0.084, Cramér’s V = 0.248). The mean scores of the HADS score for the parents from the “other sector” at time t1 were 6.33 ± 3.80 for anxiety and 3.20 ± 2.99 for depression. Regarding the parents from the “health, social services, teaching and education” sector, the scores were 7.76 ± 3.82 for anxiety and 4.41 ± 3.76 for depression.

### 3.3. Family Structure and Social Environment

There was no difference in depression between first-time mothers and those who already had children (*p* = 0.190)

Married couples showed increased “anxiety and depression syndrome” at time t1 compared to couples who were in a relationship and yet were unmarried (Fisher *p* = 0.028, Cramér’s V = 0.334). Among married couples, 21.6% developed an anxiety and depression disorder, whereas for unmarried couples in a relationship it was 0%.

**Figure 1:**
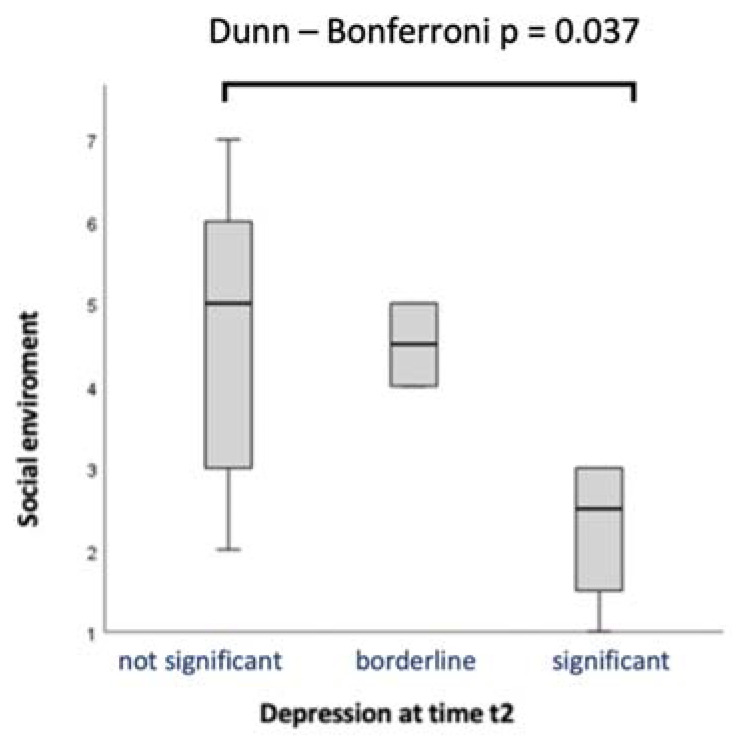
Social environment and depression.

The Kruskal–Wallis test showed a significant difference in the social environment variable between the depression classes at t2 (H [2] = 6.354, *p* = 0.042, ɛ^2^ = 0.276). The pairwise comparisons showed a significant difference between the groups “not significant” and “significant” (*p* = 0.037). Figure 1 shows that parents with a limited social environment were more likely to develop depression.

Depression at time t2 also depended on whether parents had their mothers as contacts in their environment. The percentages clearly differed between the two groups. The group without their mother as a contact person was significantly more affected (Fisher *p* = 0.035). The Cramér’s V was 0.601. This corresponds to a medium effect. If the parents had their mothers in the social environment, the HADS mean value at time t2 for depression was 3.68 ± 4.77; if not, it was 8.00 ± 6.04.

### 3.4. Gender Aspects and Pregnancy

A variable was created to further analyze the couples. For a clearer presentation, the “borderline” group and “significant” group were combined here into the “significant” group. The “not significant” group remained as such. Thus, anxiety and depression were dichotomized into “not significant” and “significant”. This resulted in the following possibilities: both partners were “significant”, one was “significant”, or both were “not significant”. If one of the partners was “significant” for anxiety and/or depression at time t1, it was typically the woman (Fisher *p* = 0.036, Cramér’s V = 0.507). Among those who suffered from an anxiety and/or depression disorder, 71.4% were women and 28.6% were men.

Regarding “pregnancy”, subjects whose pregnancies were induced by medical assistance showed a marginally significant higher anxiety score at time t1 using Fisher’s test (*p* = 0.051 Cramérs V = 0.295). In the “not significant” and borderline group, a total of 90.3% of pregnancies were not medically induced, and 9.7% resulted from medical assistance. In contrast, 35.7% of pregnancies in the “significant” group were medically induced. The HADS mean anxiety score at time t1 for pregnancies that were not medically induced was 6.68 ± 3.74, whereas that for medically induced pregnancies was 8.18 ± 4.24.

### 3.5. Results of the Hospital Anxiety and Depression Scale

The mean score for anxiety at time t1 was 6.89 ± 3.83, and at time t2 it was 7.33 ± 5.28. The mean scores for depression were 3.83 ± 3.45 at time t1 and 4.58 ± 5.23 at time t2.

Table 2 highlights the results of the “not significant”, “borderline”, and “significant” at times t1 and t2.

Anxiety and depression at t1 significantly correlated with a strong and positive effect (ρ = 0.649, *p* < 0.001). The higher the score on one scale, the higher it typically is on the other. There was also a significant and positive correlation at t2 with an even stronger effect (ρ = 0.808, *p* < 0.001) (the strength of the correlation increased from t1 to t2).

However, the depression results from t1 to t2 did not correlate significantly (ρ = 0.274, *p* = 0.195). Therefore, depression prevalence increased from pre to postnatal. Certain persons had depression at t2, although they were not depressed at t1. In contrast, anxiety scores correlated positively from t1 to t2 with a medium effect size (ρ = 0.440, *p* = 0.032). The higher the anxiety score was at time t1, the higher it was at time t2.

Table 3 provides a more detailed overview of the depressed or anxious subjects.

### 3.6. Differences between Time Points

To test for differences in depression and anxiety between the two time points, Wilcoxon tests were applied. There were no differences for anxiety (W = 100.5, *p* = 0.397, $\rho_{\mathrm{rb}}=0.330$). In addition, for depression, the Wilcoxon test showed no significant effect (W = 94.5, *p* = 0.297, $\rho_{\mathrm{rb}}=0.370)$.

### 3.7. Predictors for Mental Stress

In contrast, in logistic regression, the occupational field was meaningful. For anxiety, “borderline depression” and occupational fields (e.g., health and social workers) emerged as predictors. In all cases of at least borderline depression in time t1, “significant” anxiety at time t1 was present.

## 4. Discussion

The aim of this study was to elucidate the prevalence, correlations, and predictors of anxiety and depression in parents expecting a child with a congenital heart defect. Our study population showed a prevalence of 11.8% for anxiety and 6.6% for depressed mood at time t1. Associations were observed between depression/anxiety and the level of education and the number of contacts in the social environment. In the social environment, the mother as a person of contact had a particularly protective effect on the development of anxiety and depression. No significant association was found between the number of children, medically induced pregnancy, and occupational fields and anxiety/depression. However, positive correlations were observed between anxiety and depression scores at both study time points. The “borderline” depression and occupational sector were identified as predictors for the development of anxiety, and anxiety emerged as a predictor for depression.

Various studies have reported a relationship between education levels and anxiety or depression in the general population, specifically among pregnant women [20,21,22,23,24,25].

According to Bjelland et al., the results of their cross-sectional analysis showed a significant correlation between low educational levels and anxiety and depression, with the influence of education decreasing with increasing age. Bjelland et al. conducted both a cross-sectional analysis, and, as part of a follow-up, a longitudinal study was conducted. In the longitudinal analysis, an increase in the protective effect of the higher level of education was observed over the years. The authors attributed the different results in the two analyses to a cohort effect in the cross-sectional study. In a meta-analysis, the influence of socioeconomic status was investigated. The socioeconomic variables were education, income, occupation, social class, and assets. Again, the hypothesis was confirmed and, in addition, the results showed that individuals with a low social status were more likely to have persistent depression than recurrent episodes of depression [21]. The causes for this relationship may include lower psychosocial resources as well as the reduced use of coping strategies among people with lower levels of education [26,27]. The results of our study confirm the protective effect of a high level of education on the development of anxiety and/or depression.

Although there was a marginal association between occupational fields and anxiety/depression at time t1 in our study, the “health and social workers” occupational field acts as a predictor of anxiety. Chen et al. identified public service and health care occupations as predictors of depression during pregnancy. The high workload and stress in the healthcare industry have been discussed as causes for this, in association with an increased risk of developing anxiety and depression [28]. First, our logistic regression included a small number of cases; therefore, the results could contain subjectivity. The certain correlation with anxiety can be explained by the fact that parents working in the health sector might have more knowledge about possible negative outcomes.

According to our study, there was no difference regarding the number of children and anxiety/depression. González-Mesa et al. examined the significant associations between the number of children and the comorbidity of anxiety and depression in Turkish and Spanish populations [29]. There were more multipara women suffering from anxiety and depression in the aforementioned study. Since the study population consisted of Spanish and Turkish women, we hypothesized that cultural and religious differences, different family structures, and role distributions could lead to a higher burden of multiple children among women. In our study population, 40.3% had no children and only 3.9% had three children. This difference could also be due to the larger study population in the study by González-Mesa et al. A similar result was provided by Teixeira’s study, in which first-time mothers were more likely to have lower anxiety and depression scores in the third trimester [30]. Again, cultural differences may contribute to this finding, in addition to differences in the size of the study population, as the couples were predominantly Portuguese. A positive association between the number of completed pregnancies and conflict between partners has also been reported [31]. For parents facing the challenge of congenital heart defects, instead of conflict, there may be more cohesion and support in the partnership; therefore, the children present may not signify conflict and may not contribute to the development of anxiety or depression.

Regarding marital status, results from previous studies varied. While some studies showed no association [28,32,33], others reported a significant relationship between relationship status and mental health [34,35,36]. In particular, single status was a risk factor here. According to Adewuya et al., depression was significantly more common among people who were single, separated, divorced, or in a polygamous marriage than among those in a monogamous marriage (*p* = 0.016). Brittain et al. also reported a higher risk of maternal antenatal depression in single mothers compared with mothers living with their partner or married mothers (OR = 1.7). A comparable result was shown by Raisanen et al. There were no single mothers or separated/divorced couples in our study. Married couples were more likely to show anxiety and depression syndromes compared to unmarried couples. However, stability and satisfaction in the relationship as well as support were found to be more important than relationship statuses [28,29,31,33,37].

Across many studies, the importance of the social environment was particularly striking [29,31,33,36,37,38], as also reflected in our results. The lack of sharing about stressful situations associated with low social support, loneliness, and the reduced use of coping strategies may contribute to this observation [39]. As evident in our results and those also reported by Lee et al., the mother in particular plays an important role in the social environment [33].

Our results on the importance of gender in the development of anxiety or depression align with previous studies [30,40,41]. Hormonal changes in women during pregnancy and concerns related to childbirth are discussed as causes [42,43]. The expectations that society places on fathers and their male roles, e.g., in relation to financial stability, cause considerable stress. Fathers report insufficient involvement in the entire process, and would like to see more inclusion, support, information, and higher group representation, especially those with children with a congenital heart defect. The father–child relationship is compromised by fathers being excluded from the process and the role they must assume [44,45,46]. The anxiety of fathers and the burden they feel with their role persist even after the birth of the child in the phase of numerous operations for severe heart defects [47]. Therefore, it is possible that the pressure on fathers to display strength also led to lower levels of anxiety and depression in our study.

Kovacevic et al. examined the factors that contribute to a successful consultation after the diagnosis of a child’s heart defect [48]. Factors such as the time from diagnosis to counselling, appropriate communication and support by the counselor, and location were found to be important. The importance of time organization, communication skills, choice of words, the addition of visual materials (such as pictures and graphics), transparency, and reflection has also been reported by others [49]. In addition, it was found that parents want to have access to web-based information. Furthermore, an exchange with other affected families seems to be supportive [50,51,52].

The risk for developing perinatal anxiety appears to increase if the induction of pregnancy is supported by medical intervention [28], which was not reflected in our study. In particular, the duration of therapy and treatment failure lead to anxiety [53]. Furthermore, affected mothers seem to have greater fears of both childbirth and health problems for the child [53]. For mothers expecting a child with a heart defect, the aspect of how the pregnancy was achieved might be secondary, thus stressing the focus on the child’s disease.

If anxiety or depression is diagnosed in expecting parents, the possibility of comorbidity, both in the general population and specifically in pregnant women, requires consideration [54,55]. According to Andersson et al., anxiety and depression are present in 20.5% of women in the second trimester. Possible explanations include genetic susceptibility to both disorders and, from a neuroendocrinological perspective, elevated levels of corticotropin-releasing hormone in both disorders [56,57]. In addition, our results showed a coincidence of anxiety and depression.

## 5. Limitations

This study has several limitations. As this was a monocentric approach, there might be a selection bias. Religious, ethnic, and cultural backgrounds could influence all pregnancies and the postpartum period. Although the questionnaire was validated for pregnant women, there was no validation of the questionnaire for pregnant women expecting a child with a heart defect or other malformations. In addition, the sample size at time t2 was small. Therefore, by conducting a national multicenter approach, more general conclusions could be established.

## 6. Conclusions

The advances in fetal cardiology allow an early diagnosis of CHD and the support of parents in the post-diagnosis phase.

Our results show that a proportion of parents expecting a child with a congenital heart defect suffer from depression and/or anxiety. Considering the risk factors and predictors elaborated here, high-risk patients can now be more easily identified. Family socioeconomic factors should be analyzed in addition to a possible coincidence of anxiety and depression. Similarly, attention should also be paid to an increase in the prevalence of depression and anxiety from the prenatal to the postnatal period.

Close collaboration between maternal- fetal care units, pediatric cardiologists and psychiatric/psychosomatic disciplines, can also be used to reduce stress in parents. Therefore, an expert team of professionals educating with understandable terms and sufficient knowledge about fetal heart disease in parenting counseling is essential. The support of affected parents can positively impact the treatment of the child and should be integrated into the daily routine of the clinic.

## Figures and Tables

**Table 1:** Native language and country of birth.

		Frequency	Percentage (%)	Valid Percentage (%)	Cumulative Percentage (%)
Native language	German	66	85.7	85.7	85.7
	Other	7	9.1	9.1	94.8
	German and other	4	5.2	5.2	100
Country of birth	Germany	67	87.0	87.0	87.0
	Abroad	10	13.0	13.0	100.0

**Table 2:** Overview of the results of the HADS questionnaire.

	HADS A (n = 76)	HADS D (n = 76)	HADS A t2 (n = 24)	HADS D t2 (n = 24)
”Not significant”	53 (69.7%)	65 (85.5%)	15 (62.5%)	18 (75%)
”Borderline”	14 (18.4%)	6 (7.9%)	3 (12.5%)	2 (8.3%)
”Significant”	9 (11.8%)	5 (6.6%)	6 (25.0%)	4 (16.7%)
mean (SD)	5.97 (3.521	3.83 (3.446)	6.38 (4.871)	24 (100%)

Note: HADS A: HADS anxiety at time t1. HADS D: HADS depression at time t1. HADS A t2: HADS anxiety at time t2. HADS D t2: HADS depression at time t2.

**Table 3:** Description of the anxious or depressed subjects.

		Anxiety at t1			Depression at t1		
		Not Significant n (%)	Significant n (%)	*p*-Value *	Not Significant n (%)	Significant n (%)	*p*-Value *
Sex	Female	23 (51.1%)	22 (48.9%)	0.165	36 (80%)	9 (20%)	0.183
	Male	21 (67.7%)	10 (32.3%)		29 (93.5%)	2 (6.5%)	
Medical assisted pregnancy	No	39 (60%)	26 (40%)	0.511	57 (87.7%)	8 (12.3%)	0.192
	Yes	5 (45.5%)	6 (54.5%)		8 (72.7%)	3 (27.3%)	
Planned pregnancy	No	2 (50%)	2 (50%)	1.000	2 (50%)	2 (50%)	0.098
	Yes	42 (58.3%)	30 (41.7%)		63 (87.5%)	9 (12.5%)	
Social environment	No	0 (0%)	0 (0%)	---	0 (0%)	0 (0%)	---
	Yes	44 (57.9%)	32 (42.1%)		65 (85.5%)	11 (14.5%)	
Number of children	None	17 (54.8%)	14 (45.2%)	0.813	24 (77.4%)	7 (22.6%)	0.111
	≥ 1 child	27 (60%)	18 (40%)		41 (91.1%)	4 (8.9%)	
Surgery	No	0 (0%)	2 (100%)	0.481	1 (50%)	1 (50%)	0.342
	Yes	13 (52%)	12 (48%)		21 (84%)	4 (16%)	
Occupational field	Other	35 (68.6%)	16 (31.4%)	0.082	47 (92.2%)	4 (7.8%)	0.355
	Health, social affairs, teaching, education	7 (41.2%)	10 (58.8%)		14 (82.4%)	3 (17.6%)	
Social envirnoment including mother	No	9 (40.9%)	13 (59.1%)	0.700	18 (81.8%)	4 (18.2%)	0.721
	Yes	35 (66%)	18 (34%)		46 (86.8%)	7 (13.2%)	
Occupation	In education	0 (0%)	0 (0%)	0.087	0 (0%)	0 (0%)	0.115
	Partly employed	3 (75%)	1 (25%)		3 (75%)	1 (25%)	
	Employed	36 (59%)	25 (41%)		54 (88.5%)	7 (11.5%)	
	Self-employed	3 (100%)	0 (0%)		3 (100%)	0 (0%)	
	Unemployed	0 (0%)	3 (100%)		1 (33.3%)	2 (66.7%)	
	Unable to work	1 (100%)	0 (0%)		1 (100%)	0 (0%)	
Marital status	Married	28 (54.9%)	23 (45.1%)	0.452	40 (78.4%)	11 (21.6%)	0.013
	In a relationship	16 (66.7%)	8 (33.3%)		24 (100%)	0 (0%)	
	Single	0 (0%)	0 (0%)		0 (0%)	0 (0%)	
	Divorced	0 (0%)	0 (0%)		0 (0%)	0 (0%)	
	Widowed	0 (0%)	0 (0%)		0 (0%)	0 (0%)	
* Fisher‘s test							

## Data Availability

If required, our data can be submitted.
